# Generation and validation of a myoglobin knockout zebrafish model

**DOI:** 10.1007/s11248-023-00369-3

**Published:** 2023-10-17

**Authors:** Rasmus Hejlesen, Kasper Kjær-Sørensen, Angela Fago, Claus Oxvig

**Affiliations:** 1 Department of Biology, Zoophysiology, Aarhus University, Aarhus, Denmark; 2 Department of Molecular Biology and Genetics, Aarhus University, Aarhus, Denmark

**Keywords:** Myoglobin, Zebrafish, CRISPR/Cas, Knockout, Genetic compensation

## Abstract

Previous studies using myoglobin (*Mb*) knockout mice and knockdown zebrafish have presented conflicting results about in vivo phenotypes resulting from the loss of this conserved and highly expressed protein, and therefore a new well-characterized knockout model is warranted. We here describe the generation of three distinct zebrafish *mb* knockout lines using the CRISPR/Cas system. None of the three lines exhibited any morphological phenotypes, changes in length, or lethality during embryonic and larval development. The adult homozygous knockout *mb*(Auzf13.2) zebrafish line were absent of Mb protein, had an almost complete degradation of *mb* mRNA, and showed no changes in viability, length, or heart size. Furthermore, transcriptomic analysis of adult heart tissue showed that *mb* knockout did not cause altered expression of other genes. Lastly, no off-targeting was observed in 36 screened loci. In conclusion, we have generated three *mb* knockout lines with indistinguishable phenotypes during embryonic and larval development and validated one of these lines, *mb*(Auzf13.2), to have no signs of genetic compensation or off-target effects in the adult heart. These findings suggests that the *mb*(Auzf13.2) shows promise as a candidate for investigating the biological role of Mb in zebrafish.

**Supplementary Information:**

The online version contains supplementary material available at 10.1007/s11248-023-00369-3.

## Introduction

Myoglobin (Mb) functions as an intracellular O_2_ carrier and storage protein, highly expressed in vertebrate heart and skeletal muscle (Wittenberg and Wittenberg 2003). The in vivo function of Mb has been the topic of many studies using several different mouse knockout (KO) models (Garry et al. 1998; Gödecke et al. 1999; Park et al. 2019; Ono-Moore et al. 2021), and one zebrafish knockdown (KD) model (Vlecken et al. 2009) (Supp. Figure 1). However, the in vivo findings have been contradictory (Supp. Table 1). Initial studies in mice showed no apparent effects of the absence of Mb, except for reduced coloring of the heart and the soleus muscle (Garry et al. 1998). Later, it was reported that most *Mb*^(−/−)^ mice die in utero with signs of cardiac failure (Meeson et al. 2001), whereas a recent study found normal survival rate of *Mb*^(−/−)^ mice (Ono-Moore et al. 2021). In assessments of endurance exercise with high O_2_ demand, one study found a decline (Merx et al. 2005), while another study observed no changes in endurance of *Mb*^(−/−)^ mice (Garry et al. 1998). Reports on heart size in adult *Mb*^(−/−)^ mice are also conflicting, with studies indicating no change (Gödedke et al. 2003; Merx et al. 2005; Hendgen-Cotta et al. 2008; Ono-Moore et al. 2021), and one reporting a decreased heart size (Hendgen-Cotta et al. 2017). A consequence of Mb loss in mice may involve reducing O_2_ consumption in heart and skeletal muscle by shifting substrate utilization from fatty acids to carbohydrates. In the hearts of *Mb*^(−/−)^ mice, increased glucose (Flögel et al. 2005) and lactate (Meeson et al. 2001) utilization has been reported. Flögel et al. (2005) observed a significant decrease in fatty acid utilization, while Meeson et al. (2001) only noted a non-significant trend. This shift from fatty acids is expected to result in an increased respiratory exchange ratio (RER). However, conflicting findings have emerged regarding changes in the RER in *Mb*^(−/−)^ mice, with Merx et al. (2005) reporting an increased RER, whereas Ono-Moore et al. (2021) and Christen et al., (2022) did not observe this. Lastly, the recent study by Ono-Moore et al. (2021) did not report a transition from oxidative to non-oxidative muscle types as reported earlier in mice (Grange et al. 2001). One finding that is consistent between studies of *Mb*^(−/−)^ mice is the increase in heart vascularity (Gödecke et al. 1999; Meeson et al. 2001; Mammen et al. 2003). In zebrafish, a morpholino *mb* knockdown model showed increased lethality, body curvature and heart defects (Vlecken et al. 2009). Taken together, these findings have failed to produce a consistent picture of how Mb functions in vivo, and whether compensation at various levels may occur or not.

Several factors might explain these contradictory findings. Different species and strains may show variable adaptation in the absence of Mb. Additionally, the method used to generate KO and KD models can result in unintended off-target effects. For instance, the Mb KO models (Garry et al. 1998; Gödecke et al. 1999) were made by replacing exon 2 with a neo selection cassette (Supp. Figure 1), which potentially interferes with expression of other genes (Pham et al. 1996; Scacheri et al. 2001; Meier et al. 2010). The risk of morpholino off-target effects has also been documented (Kok et al. 2015), and earlier studies, e.g. Vlecken et al. 2009, may not follow the best practice of today (Stainier et al. 2017). To the best of our knowledge, neither of the Mb KO models have considered the recent concept of genetic compensation (GC) (El-Brolosy et al. 2019; Ma et al. 2019). In short, GC is a process where degraded mRNA may result in the upregulation of similar genes. The common strategy of introducing an early frame shift to generate a KO model may trigger GC. Two other approaches can be used to avoid GC: creating RNAless mutants by deleting the promoter region or the entire locus, or generating in-frame deletions that lead to the expression of a non-functional protein (Sztal and Stainier 2020). However, larger deletions may cause the removal of regulatory elements, and translated non-functional protein may give rise to unwanted side effects.

Here, we generated three genetically distinct *mb* KO zebrafish lines using two different CRISPR/Cas approaches. All lines showed identical embryonic and larval development, indicating that none of the lines show any GC or off-target effects. One of the established lines was further validated using transcriptomics to confirm the lack of GC and off-target effects. We are confident that these lines will facilitate future studies of the in vivo function of Mb in zebrafish.

## Methods

### Zebrafish husbandry and ethics

Zebrafish were breed in-house using AB founders from the European Zebrafish Resource Center. Fish were fed four times daily and maintained on a 14-h-light–10-h-dark cycle on recirculating housing systems at 28 °C. Embryos were obtained by natural crosses, reared in E3 buffer [5 mM NaCl, 0.17 mM KCl, 0.33 mM CaCl2, 0.33 mM MgSO_4_, 10^−5^% (w/w) methylene blue, 2 mM HEPES pH 7.2] at 28 °C. All experiments were carried out according to Danish legislation and approved by The Danish Animal Ethics Council (permit number 2017-15-0201-01380/2023-15-0201-01448).

### gRNA design and testing

Potential gRNAs were predicted in silico using CHOPCHOP v. 3 (Labun et al. 2019), CRISPRscan (Moreno-Mateos et al. 2015), and CRISPOR (Concordet and Haeussler 2018), targetting the singular *mb* gene in zebrafish (GRCz11 annotation, and no similar nucleotide or protein hits were found using BLAST). Based on predicted efficiency, few off-targets, and overlap between the different algorithms, we tested 19 gRNAs. Oligos were designed according to (Wang et al. 2014) (Supp. Table 2). The pDR274 plasmid (Addgene, #42,250) was linearized with *BsaI* (Thermo Scientific, #ER0292), separated on an agarose gel, and purified with GenElute™ Gel Extraction Kit (Sigma-Aldrich, #NA1111). Oligos were annealed and ligated into linearized pDR274. The purified plasmids were linearized by *DraI* (Thermo Scientific, #ER0223), and gRNA were transcribed and purified using the T7 MEGAshortscript kit (Thermofisher, #AM1354).

The yolk of wild-type zebrafish embryos were microinjected no later than at the 1-cell stage with 5 nL injection mixture (392 ng/µL Cas9 mRNA, 400 ng/µL gRNA, 0.125 M KCl, and phenol red). For each gRNA tested, 10 embryos were dechorionated at 24 h post fertilization (hpf), euthanized in excess tricaine, and fixed in 100% MeOH. MeOH was removed before the embryos were dissolved in 100 µL TE buffer containing 1.7 mg/mL proteinase K (Ambion, #AM2548) for 2–4 h at 55 °C. When embryos were completely dissolved, proteinase K was deactivated by incubation at 95 °C for 5 min. 1 µL of the dissolved embryos were used as template in a 25 µL PCR reaction using the KOD Hot Start DNA polymerase kit (Sigma-Aldrich, #71,086). For gRNA 1–10, a 1988 bp amplicon spanning exon 1 of *mb* was generated using the primer pair mbEx1-1240_f/mbEx1 + 728_r (Supp. Table 3) with the following program: 1 × 95 °C for 2 min; 35 cycles of 20 s at 95 °C, 10 s at 61 °C, 1 min and 45 s at 68 °C. For gRNAs 11–14 + 16–19 + 21, a 474 bp amplicon spanning exon 2 of *mb* was generated using the primer pair mbEx2-202_f/mbEx2 + 272_r (Supp. Table 3) with the following program: 1 × 95 °C for 2 min; 35 cycles of 20 s at 95 °C, 10 s at 61 °C, 1 min at 68 °C. The amplicons were Sanger sequenced using primer mbEx1 + 482_r and mbEx2-202_f, respectively (Supp. Table 3). To estimate the in vivo efficiencies the sequencing results were analyzed using TIDE and ICE (Brinkman et al. 2014; Tim et al. 2018).

### Generating KO lines

For generation of KO lines, microinjections were done similarly. For line *mb*^Auzf13.2^ and *mb*^Auzf13.6^, the injection mixture was 392 ng/µL Cas9 mRNA, 100 ng/µL #4 gRNA, 100 ng/µL #7 gRNA, 100 ng/µL #12 gRNA, 100 ng/µL #21 gRNA, 0.125 M KCl, and phenol red. For line *mb*^Auzf13.2^, the following injection mixture was used: 392 ng/µL Cas9 mRNA, 400 ng/µL #13 gRNA, 175 ng/µL repair template, 0.125 M KCl, and phenol red. At 90 + days post fertilization (dpf), the potential founders (F0) were out-crossed with AB wild-type zebrafish. Sixteen resulting embryos were genotyped by Sanger sequencing. Promising F0 were out-crossed with AB wild-type and allowed to mature before genotyping, resulting in the establishment of heterozygous F1 mutants (*mb*^Auzf13.X±^). Generation of F2, F3, etc. were done by out-crossing with AB wild-type. F2 or F3 *mb*^Auzf13.x(±)^ were in-crossed to generate F2 or F3 homozygous for the respective mutation *mb*^Auzf13.x(−/−)^, heterozygous for the mutation *mb*^Auzf13.x(±)^, or homozygous without the mutation F2 *mb*^Auzf13.x(+/+)^ (Supp. Figure 2).

### Genotyping

Genotyping was performed by using caudal tail fin clips, or 24–48 hpf whole embryos. The caudal tail fin was cut from 90 + dpf zebrafish, anesthetized in tricaine (170 mg/L). The Phire™ Tissure Direct PCR kit (ThermoFisher, #F170S) was used for both caudal tail fins and embryos, following the manufacturer’s protocol. A 1343 bp amplicon spanning exon 1 and exon 2 of *mb* was generated using the primer pair mbEx1-282_f/mbEx2 + 353_r (Supp. Table 3) with the following program: 1 × 98 °C for 5 min; 40 cycles of 5 s at 98 °C, 5 s at 58 °C, and 30 s at 72 °C ending with a final elongation at for 60 s at 72 °C. Initially, the amplicons were forward and reverse Sanger sequenced using mbEx1-205_f or mbEx2 + 266_r, respectively (Supp. Table 3). Later, amplicon size difference was used to separate *mb*^(+/+)^, *mb*^(/-)^, and *mb*^(−/−)^ from the *mb*^Auzf13.3^ and *mb*^Auzf13.6^ lines. The small insertion in line *mb*^Auzf13.2^ required digestion with *BcnI* (ThermoFisher, #FD0064) for separation of *mb*^(+/+)^, *mb*^(/-)^, and *mb*^(−/−)^. At 6 dpf, 371, 252, and 272 embryos were genotypes for *mb*^Auzf13.2^, *mb*^Auzf13.3^, and *mb*^Auzf13.6^, respectively. A total of 98 adult *mb*^Auzf13.2^ were genotyped.

### Developmental phenotyping, heart and length measurement

Blinded phenotyping was performed daily on offspring from in-crossed F2 or F3 *mb*^Auzf13.2^ (n = 253), *mb*^Auzf13.3^ (n = 252), and *mb*^Auzf13.6^ (n = 272) during the first 6 dpf. Developmental stage was determined by counting somites at ~ 22 hpf (Kimmel et al. 1995). At 6 dpf, larvae was anesthetized in E3 containing tricaince (0.15 g/L). Images were taken of embryos using an OLYMPUS SZX16 microscope equipped with a ZEISS Axiocam 305 color camera. Larval standard length was measured using the ZEN core 3.4 software (Parichy et al. 2009). At 6 dpf, 196, 252, and 272 larvae were measured for *mb*^Auzf13.2^, *mb*^Auzf13.3^, and *mb*^Auzf13.6^, respectively. Following imaging, the larvae were euthanized and genotyped. Images of adult *mb*^Auzf13.2^ zebrafish (n = 77) were taken on a mm grid to allow determination of standard length using the ImageJ software (Parichy et al. 2009). Hearts (n = 30) were dissected, imaged, and cross-section measured using the same setup as for larvae.

### Western blotting

Ventricles (7–13 ventricles weighing a total of 2.74–4.31 mg) were pooled in 150 µL 100 mM HEPES, pH 4, with 2.5 mM EDTA. The samples were homogenized using Precellys Evolution in 0.5 mL tubes with ceramic beads type CK14 (VMR, #432-0375P). Protein concentrations (0.17–0.36 mg/mL) were determined using Pierce™ 660 nm Protein Assay Reagent (ThermoFisher, # 22,660), following the manufacturer’s protocol.

Samples of heart homogenate (2.28, 1.53, or 0.765 µg total protein) of *mb*^Auzf13.2(+/+)^ and *mb*^Auzf13.2(−/−)^ were separated on a 12% ExpressPlus Page gel (GenScript, #M01212). The proteins were blotted onto a PVDF membrane (Merck Milipore) followed by blocking in 2% Tween-20 in TST (50 mM TRIS, 0,5 M NaCl, 0.1% Tween-20, pH 9.0) for 5 min. The membrane was incubated for 16 h at 4°C with primary polyclonal Mb antibody (LS-C144978-200), diluted 1:1000 in TST containing 2% skimmed milk powder, and then incubated for 1 h at RT using secondary polyclonal anti(rabbit IgG)-HRP (DAKO, P0217) diluted 1:2000 in TST with 2% skimmed milk powder. Between all steps, the membrane was washed three times with TST. The blot was developed using enhanced chemiluminescence (ECL Prime, GE Healthcare) followed by image capturing on an ImageQuant LAS 4000 instrument (GE Healthcare).

### RNA purification, sequencing, and data analysis

Three freshly dissected ventricles from adult *mb*^Auzf13.2^ were pooled per replicate (0–1 females and 2–3 males). Four replicates were made for the *mb*^Auzf13.2(−/−)^ and *mb* ^Auzf13.2(+/+)^ genotypes. RNA was isolated and purified using the RNeasy Micro kit (Qiagen, #74,004). Quality control and sequencing was carried out by Novogene (UK). Reads were aligned to the GRCz11 reference and gene model (Ensembl release 109) using STAR 2.7.10b (Dobin et al. 2013). Aligned fragments (paired-end read pair) were counted using FeatureCounts (Liao et al. 2013). Finally, differential gene expression were determined using DESeq2 (Love et al. 2014) comparing the *mb*^(−/−)^ with *mb*^(+/+)^.

STAR aligned reads were visualized in the Integrative Genomics Viewer (Robinson et al. 2011). CRISPRoff (Anthon et al. 2022) was used to generate a list of potential off-targets of gRNA4, 7, 12, and 21 in the danRer11 genome. The list was filtered for off-targets in transcribed regions with a read coverage of at least 10x. The filtered lists were manually screened for INDELs 1 kb up- and downstream from potential off-targets if coverage would allow.

### Data analysis

The galaxy platform (v 23.0) was utilized for alignment, counting, and differential gene expression analysis (Community 2022). One-way Anova, X^2^, and exact Fisher’s test were performed in R using stats (v 4.3.1). All graphs were produced in R using the tidyverse package (v 2.0.0) (Wickham et al. 2019).

## Results and discussion

Nineteen gRNAs targeting the *mb* gene in zebrafish were designed in silico and co-injected individually with Cas9 mRNA into 1-cell zebrafish embryos. At 24 hpf, the *mb* gene was amplified using PCR and Sanger sequenced to estimate in vivo efficiency by using TIDE and ICE (Brinkman et al. 2014; Tim et al. 2018) (Supp. Table 4).

**Fig. 1:**
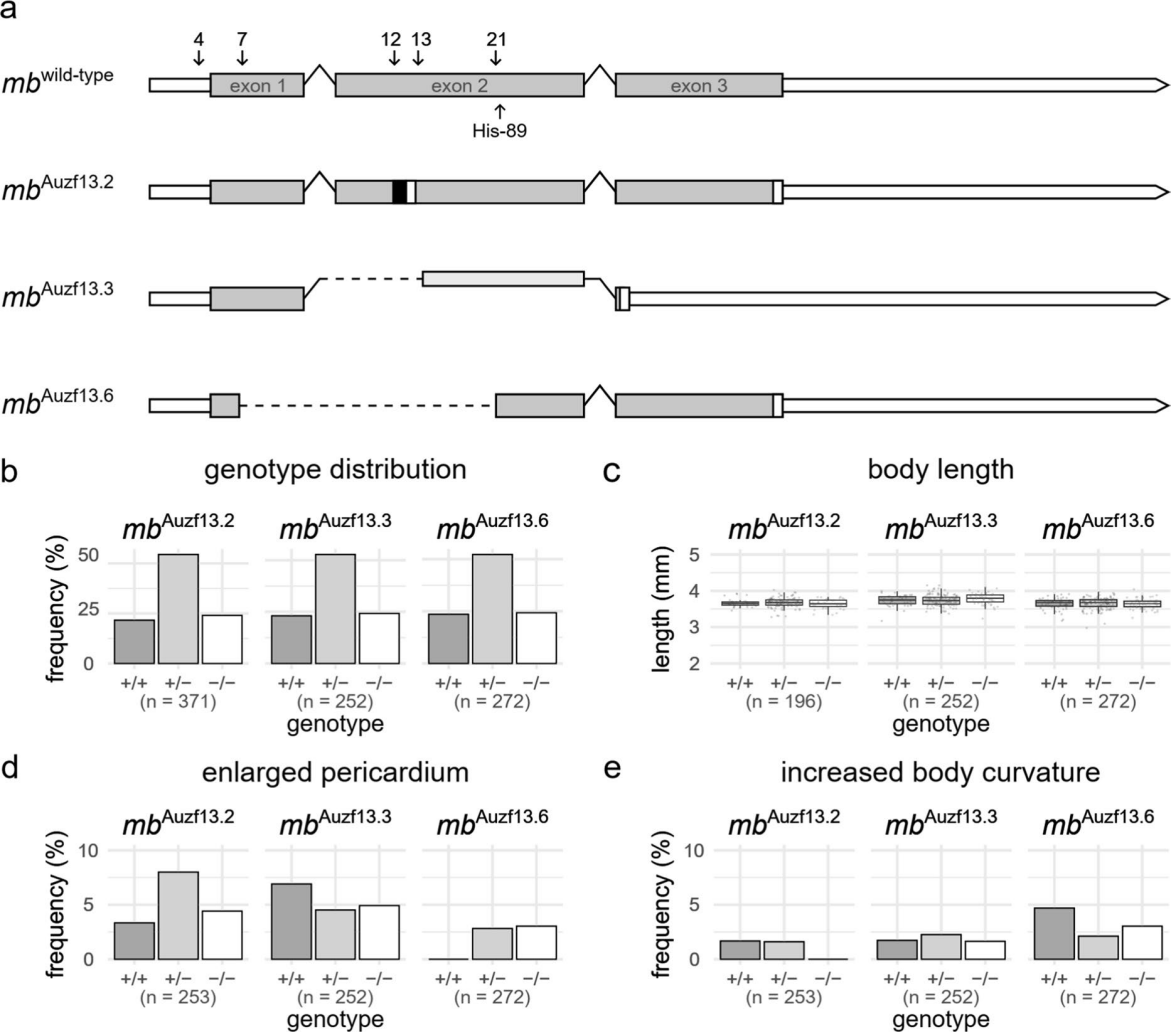
Generated myoglobin (*mb*) knockout lines, embryonic body length and genotype distribution. **a** Schematic representation of the zebrafish *mb* gene (top) and the three variants generated in this study. Exons are shown as grey bars, introns as black lines, and stop codon and UTR are white bars. The positions of guide RNAs and His-89 are indicated by numbered arrows and text, respectively. For generation of the *mb*^Auzf13.2^ and the *mb*^Auzf13.6^ lines, gRNAs 4, 7, 12, and 21 were used. For generation of the *mb*^Auzf13.2^ gRNA 13 was used. Insertions are shown as a black bar in *mb*^Auzf13.2^. Deletions are shown as dotted lines in *mb*^Auzf13.3^ and *mb*^Auzf13.6^. **b** Genotype frequency at 6 dpf from F2 or F3 *mb*^(±)^ in-crosses. Using an X^2^-test, no statistically significant deviation from the expected Mendelian distribution in *mb*^Auzf13.2^ (X^2^ = 3.372, df = 2, *p* = 0.1853), *mb*^Auzf13.3^ (X^2^ = 0.84921, df = 2, *p* = 0.654) or *mb*^Auzf13.6^ (X^2^ = 0.55882, df = 2, *p* = 0.7562) was found. **c** Length measurements of the three KO lines at 6 dpf from F2 or F3 *mb*^(/-)^ in-crosses. Using a one-way ANOVA test, no statistically significant difference in average length according to genotype for *mb*^Auzf13.2^ (F(2) = 1.012, *p* = 0.366), *mb*^Auzf13.3^ (F(2) = 1.932, *p* = 0.147) or *mb*^Auzf13.6^ (F(2) = 0.128, *p* = 0.879) was found. **d** Frequency of enlarged pericardium at 6 dpf from F2 or F3 *mb*^(/-)^ in-crosses. Using a Fisher’s exact test, no statistically significant difference in occurrence of enlarged pericardium in *mb*^Auzf13.2^ (*p* = 0.4747), *mb*^Auzf13.3^ (*p* = 0.7556), or *mb*^Auzf13.6^ (*p* = 0.5524) was found. **e** Frequency of deviation from normal body curvature. Using a Fisher’s exact test, no statistically significant deviation from normal body curvature in *mb*^Auzf13.2^ (*p* = 0.6126), *mb*^Auzf13.3^ (*p* = 0.6126), or *mb*^Auzf13.6^ (*p* = 0.5794) was found

To generate potential KO founders (F0), two distinct approaches were employed. Line *mb*^Auzf13.2^ and *mb*^Auzf13.6^ were generated using gRNAs 4, 7, 12, and 21 together, whereas line *mb*^Auzf13.3^ was generated using gRNA 13 (Fig. 1a, Supp. Table 2), as detailed in Methods. The first line, *mb*^Auzf13.2^, has a 1 bp deletion (C) and a 6 bp insertion (GGTGGT), resulting in a frameshift and a premature termination codon (Fig. 1a, Supp. Table 5–6), expected to lead to nonsense mediated decay (NMD), and potentially GC. The frameshift in *mb*^Auzf13.2^ occurs before His-89 (positioned in helix F8), involved in heme binding, causing any translated protein to be dysfunctional (Supp. Table. 7). The second line, *mb*^Auzf13.3^, carries a large deletion (438 bp) spanning the acceptor splice site of exon 2 in the *mb* gene (Fig. 1a, Supp. Table 5), most likely resulting in transcription of mRNA lacking exon 2 (Supp. Table 6). The splicing of exon 1 and exon 3 will lead to a frameshift in exon 3. The absence of exon 2 will result in non-functional protein, as His-89 is located within this exon (Supp. Table 7). Because exon 3 contains the native termination codon, it is not expected that the introduction of a premature termination codon will result in NMD, thus decreasing the risk of GC. The third line, *mb*^Auzf13.6^, carries a large (856 bp) in-frame deletion, resulting in the splicing of exon 1 and exon 2 (Fig. 1a, Supp. Table 5–6). Similarly, any translated protein will not be functional due to the lack of His-89 (Supp. Table. 7). In the absence of NMD, it is unlikely that the modification of the *mb*^Auzf13.6^ line will result in GC.

To characterize the three *mb* KO lines, we in-crossed heterozygous F2 or F3 to allow blinded screening for viability, length, and any morphological/developmental defects during the first six days of development before genotyping. We observed the expected Mendelian ratios at 6 dpf in all three lines (Fig. 1b). Furthermore, we found no significant difference in length, according to genotype for any of the three lines (Fig. 1c). Lastly, we observed no genotype specific changes in the frequency of enlarged pericardium (Fig. 1d), or increased body curvature (Fig. 1e). In contrast, an earlier study based on *mb* morpholino knockdown in zebrafish, reported an enlarged pericardium, increased body curvature, and increased lethality (Vlecken et al. 2009).

**Fig. 2:**
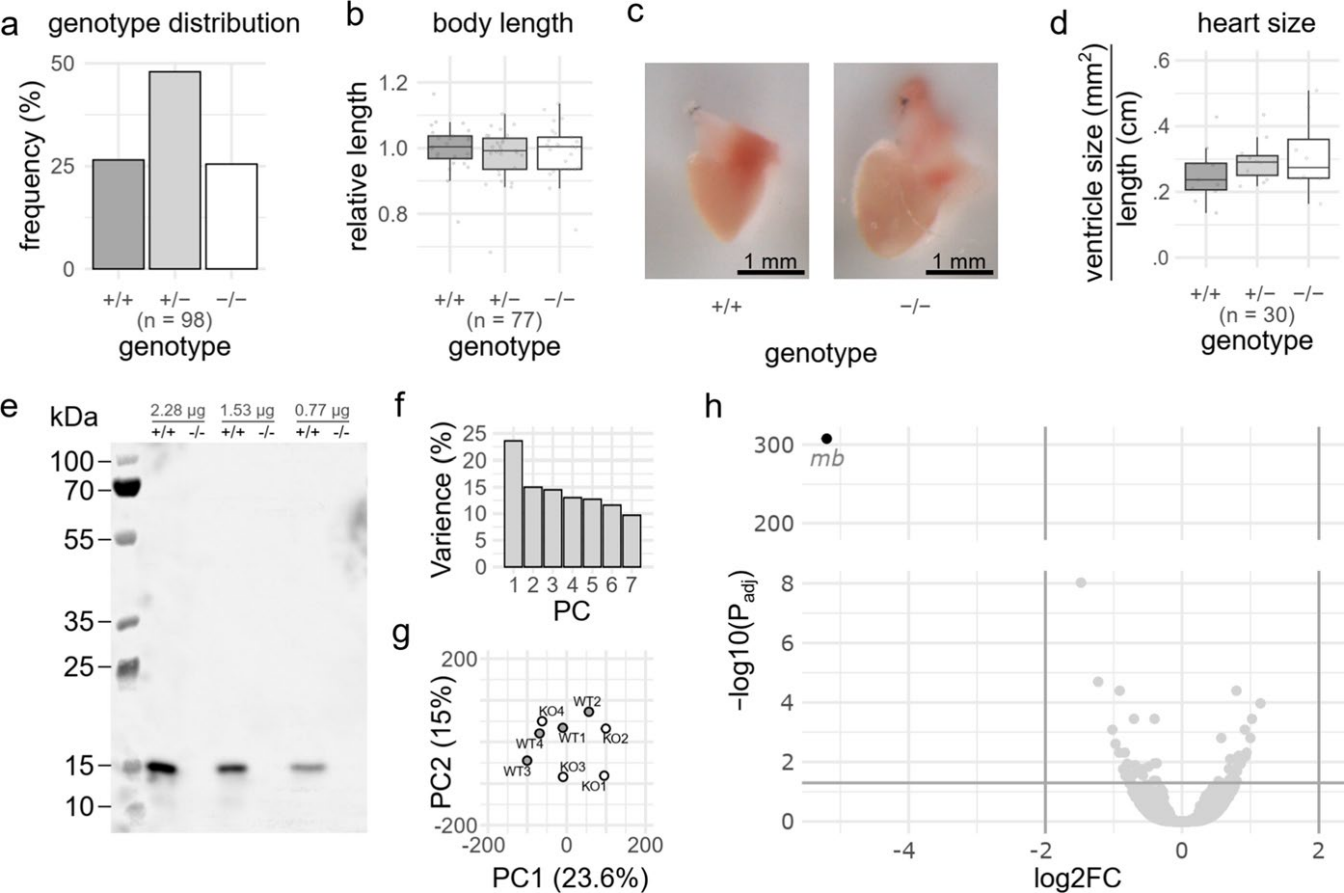
Characterization of adult (3–6 months post fertilization) *mb*^Auzf13.2^ zebrafish resulting from in-crossed *mb*^Auzf13.2(±)^. **a** Genotype frequency distribution. Using an X^2^-test, no statistically significant deviation from the expected Mendelian distribution (X^2^ = 0.18367, df = 2, *p* = 0.9123) was found. **b** Zebrafish length relative to mean length of *mb*^Auzf13.2(+/+)^ siblings. Using a one-way ANOVA test, no statistically significant difference in length according to genotype (F(2) = .474, *p* = 0.624) was found. **c** Adult hearts from wild-type and knockout (*mb*^Auzf13.2(−/−)^) fish. **d** Ventricle size measured by its cross section. Using a one-way ANOVA test, no statistically significant difference in ventricle cross section area normalized to standard length when comparing genotypes (F(2) = 1.12, *p* = 0.341) was found. **e** Mb specific western blot of heart homogenate from the *mb*^Auzf13.2^ with the wild-type (+ / +) and knockout (−/ −) genotype. Three different protein amounts (left to right: 2.28, 1.53, and 0.765 µg) of total protein were loaded. Principal component (PC) analysis of gene counts from RNA sequencing comparing knockout vs. wild-type zebrafish heart ventricles. **f** Scree plot for all PCs. **g** Plot of PC1 and PC2. **h** Volcano plot depicting log2 fold change (FC) in expression and the − log10(P_adj_) values. Horizontal grey line indicated a P_adj_ value of 0.05. The vertical line depict log2FC of − 2 and 2

For further characterization, we chose the *mb*^Auzf13.2^ line as its limited nucleotide change makes it least likely to disrupt hypothetical genetic regulatory elements. F2 or F3 heterozygous *mb*^Auzf13.2^ were in-crossed and allowed to grow to adulthood. We observed no genotype specific changes in Mendelian distribution (Fig. 2a), length (Fig. 2b), or ventricle size (Fig. 2c-d) in the adult *mb*^Auzf13.2^ zebrafish. Altogether, these findings indicate no change in development of the *mb*^Auzf13.2(−/−)^ fish compared to wild-type fish. We did not observe a change in coloring of the heart by visual inspection (Fig. 2c) as seen in mice (Garry et al. 1998; Gödecke et al. 1999), possibly due to the presence of residual blood in the spongious zebrafish heart, as compared to the more compact mammalian heart. Nevertheless, the absence of Mb in heart muscle of *mb*^Auzf13.2(−/−)^ fish was confirmed by western blotting (Fig. 2e).

To assess possible unwanted genomic changes in the *mb*^Auzf13.2(−/−)^ line, we screened 36 in silico predicted potential off-target sites located in transcribed regions with sufficient read coverage (Table 1). Except for one deletion, located ~ 350 bp from a potential off-target, we did not observe any deviation compared to the reference genome (Supp. Figure 3). Based on the large distance between the predicted off-target site and the observed deletion, it is most likely not a consequence of the editing procedure. The observed deletion is located in the untranslated region (UTR) of the *igf1rb* gene, thus unlikely to have any effect. The deletion is located on a different chromosome than *mb*, and is probably carried by our wild-type strain (AB).

**Table 1:** Assessment of modification at predicted off-target sites

Critical off-target	gRNA4	gRNA7	gRNA12	gRNA21
All	2	318	20	7
Gene	1	189	14	4
Exon	0	30	5	1
INDELs	NA	1/30	0/5	0/1

The total number of critical off-targets as predicted by CRISPR-off (Anthon et al. 2022) for the four gRNAs used for generation of the *mb*^Auzf13.2^ zebrafish line is shown in the All row. The following two rows show the number of off-targets in genes and exons, respectively. The last row shows the number of INDELs found within 1 kb of the predicted off-target sites

Finally, to assess potential changes in transcription, RNA from adult ventricles of *mb*^Auzf13.2(+/+)^ and *mb*^Auzf13.2(−/−)^ were sequenced and analyzed. 85–93% of the reads obtained (> 20 million reads for each of eight samples) uniquely mapped to the reference genome using STAR (Dobin et al. 2013). Comparing gene counts using principal component analysis showed a surprisingly high degree of similarity between the *mb*^Auzf13.2(+/+)^ and *mb*^Auzf13.2(−/−)^ lines (Fig. 2f-g). Apparently, the only significant differentially expressed gene with a log2FC lower than -2 or higher than 2 was *mb* (log2FC = -5.20) (Fig. 2h, Supp. Table. 8), when using DESeq2 (Love et al. 2014). The mutated *mb* gene showed a 37-fold reduction, reflecting its almost complete degradation.

The similarity between gene expression in *mb*^Auzf13.2(+/+)^ and *mb*^Auzf13.2(−/−)^ makes it safe to assume that the lack of observed phenotypes is not a result of GC. Furthermore, it is worth pointing out that none of the transcriptional adaptations seen in Mb KO mice, such as increased HIF-1 and HIF-2 expression (Grange et al. 2001; Meeson et al. 2001), are present in the heart of the *mb*^Auzf13.2^ line.

## Conclusion

We successfully generated three distinct KO zebrafish lines for *mb* using the CRISPR/Cas system. Although these lines carried different mutations, all were expected to result in disruption of Mb function, either by the disruption of translation, or by the translation of a non-functional Mb protein. There were no significant changes in viability, embryo length, or other morphological phenotypes during the initial six days of development in all lines. Among the three lines, we selected the *mb*^Auzf13.2^ line, which carried a mutation least likely to interfere with regulatory elements, for further validation. We confirmed the absence of functional *mb* mRNA and of Mb protein in heart ventricles of the *mb*^Auzf13.2(−/−)^ line. Additionally, we screened 36 potential CRISPR/Cas off-targets and found no mutations at these sites. Moreover, there were no alterations in viability, standard body length, or heart size in adult *mb*^Auzf13.2^. The removal of Mb did not lead to any appreciable changes in the expression of other genes, indicating no GC mechanisms in the *mb*^Auzf13.2^ line. Based on these findings, we are confident that the *mb*^Auzf13.2^ KO line has undergone sufficient validation to warrant further functional analysis. This line holds potential for elucidating the in vivo function of the *mb* gene in zebrafish.

### Supplementary Information

Supplementary file 1 (PDF 645 KB)

## Data Availability

The data that support the findings of this study are available from the corresponding author upon reasonable request.
