# Supplementary material for: Generation and validation of a myoglobin knockout zebrafish model
Source: Transgenic Res. 2023 Oct 17;32(6):537–46. doi: 10.1007/s11248-023-00369-3 (PMC10713697; doi:10.1007/s11248-023-00369-3)
Supplement: Supplementary file 1 — (PDF 645 KB) [file 11248_2023_369_MOESM1_ESM.pdf]

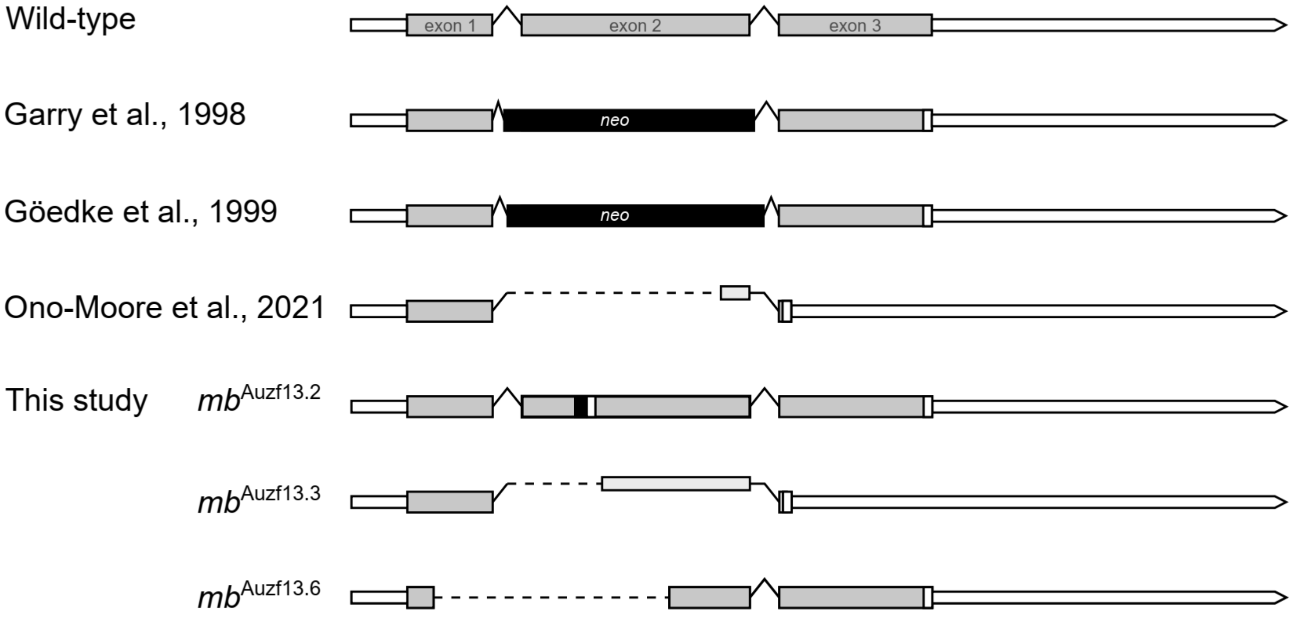

Supp. Fig. 1. Myoglobin knockout mice and zebrafish models. Schematic representation of the wild-type mouse and zebrafish myoglobin gene and the six existing knockout models (including the three generated in this study). The locus is simplified to look identical for mice and zebrafish, both species consist of 3 coding exons with the distal and proximal histidine located in exon 2. Exons are shown as grey bars, introns as black lines, and stop codon and UTR are white bars. Insertions are shown as a black bar in Garry et al., 1998, Göedke et al., 1999 and the *mb*$^{\text{Auzf13.2}}$ models representing the *neo* selection cassette for the former two and 5bp net insertion for the latter. Deletions are shown as dotted lines in the Ono-Moore et al., 2021, *mb*$^{\text{Auzf13.3}}$, and *mb*$^{\text{Auzf13.6}}$ models

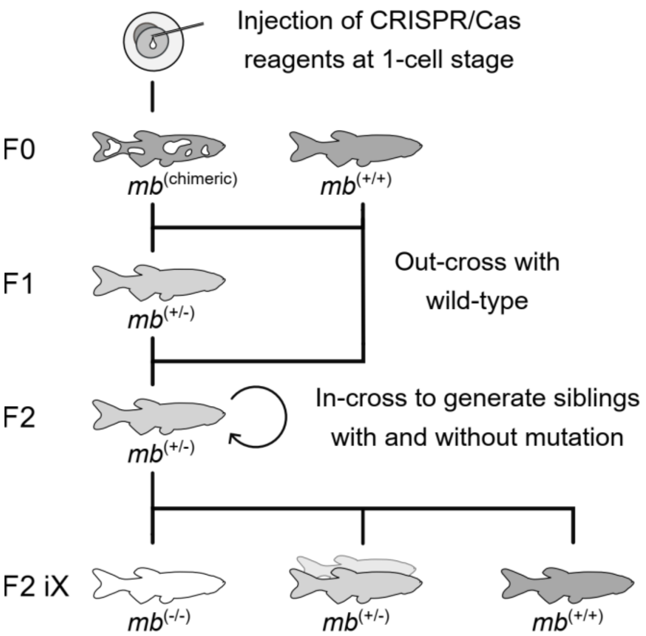

Supp. Fig. 2. Generation of zebrafish lines. Embryos were injected with CRISPR/Cas reagents at the 1-cell stage, resulting in the generation of chimeric founders. Heterozygous (*mb*$^{(+/-)}$) F1 fish were generated by out-crossing of the chimeric F0 fish with wild-type fish (*mb*$^{(+/-)}$). Further out-crossing generating new generations (F2, F2, etc.). In-crossing of heterozygous F2 or older will result in a Mendelian distribution of 25% *mb*$^{(-/-)}$, 50% *mb*$^{(+/-)}$, and 25% *mb*$^{(+/+)}$ under the assumption that the survival rate of mutant and wild-type is similar. In-crossing in this way ensures we get mutant and wild-type sibling for experimentation

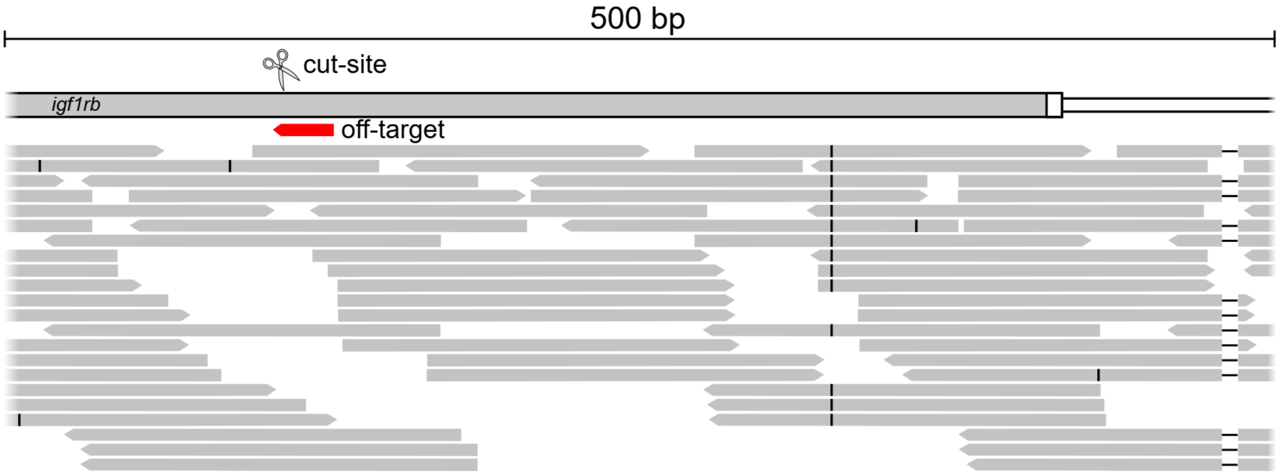

Supp. fig. 3 Off-target screening. We show a zoom in on the *igf1rb* gene, the grey bar indicating the last exon, the white square indicating the native termination codon, and the thin white bar indicates the 3'UTR. The predicted off-target region is marked as a red arrow and a black arrow indicates the off-target cut site. Aligned read are shown as grey arrows with the pointed end indicating sequencing direction. We observed a deletion ~350 bp downstream from the potential off-target, indicated by a thin horizontal black line in all reads. Sequencing mismatches is shown as thin vertical black lines in individual read, indicating SNPs or sequencing mistakes

Supp. Table 1 Overview of existing myoglobin knockout and knockdown models and reported phenotypes. ↑ significant increase in knockout/knockdown as compared to wild-type. ↔ no significant difference between knockout/knockdown as compared to wild-type. ↓ significant decrease in knockout/knockdown as compared to wild-type. HFD: mouse were fed on a high fat diet before comparing knockout with wild-type. – indicates that the information is not reported. NA indicates that the information is not applicable for this method. LV: left ventricle.

Model origin	**Mouse knockout by Garry et al., 1998**	**Mouse knockout by Göedke et al., 1999**	**Mouse knockout by Ono-Moore et al., 2021**	**Zebrafish knockdown by Vlecken et al., 2009**	**Zebrafish knockout current study**
**Species**	*Mus musculus*	*Mus musculus*	*Mus musculus*	*Danio rerio*	*Danio rerio*
**Strain**	– (Garry et al. 1998)<br>– (Grange et al. 2001)<br>C57BL/6J (Meeson et al. 2001)<br>Sv 129 (Meeson et al. 2001)	NMRI	C57BL/6J	–	AB
**Method**	Selection cassette, knockout	Selection cassette, knockout	CRISPR/Cas, knockout	Morpholino, knockdown	CRISPR/Cas, knockout (3x)
**Off-targets (found/screened)**	0/0	0/0	0/7	NA	0/36
**Genetic compensation**	Not assessed	Not assessed	Not assessed	NA	Not detected
**Insertion/deletion**	neo cassette/exon2	neo cassette/exon2 (1.2kb)	0/199 bp	NA	6/1 bp
**Embryonic**					
Survival rate	↓ (Meeson et al. 2001)		↔ (Ono-Moore et al. 2021)	↓ (Vlecken et al. 2009)	↔
Size	↓ development (Meeson et al. 2001)				↔ length
Heart failure	↑ (Meeson et al. 2001)			↑ (Vlecken et al. 2009)	↔
Curvature defect				↑ (Vlecken et al. 2009)	↔
**Adult**					
Survival rate	↓ (Meeson et al. 2001)		↔ (Ono-Moore et al. 2021)		↔
Animal size	↔ mass (Garry et al. 1998)	↔ mass (Flögel et al. 2005)<br>↔ mass (Hendgen-Cotta et al. 2008)<br>↔ mass (Hendgen-Cotta et al. 2017)<br>↓ mass/length (Christen et al. 2022)	↔ mass on HDF (Ono-Moore et al. 2021)		↔ length
Heart pigmentation	↓ (Garry et al. 1998)	↓ (Gödecke et al. 1999)			↔
Type I to II muscle transition	↑ (Grange et al. 2001)		↔ on HFD (Ono-Moore et al. 2021) (HFD)		
Depigmentation	↓ (Garry et al. 1998)	↓ (Gödecke et al. 1999)			
Heart size		↔ (Gödecke et al. 2003)<br>↔ (Merx et al. 2005)<br>↔ (Hendgen-Cotta et al. 2008)<br>↓ LV mass (Hendgen-Cotta et al. 2017)	↔ on HDF (Ono-Moore et al. 2021)		↔
Heart vasculature	↑ (Meeson et al. 2001)<br>↑ (Mammen et al. 2003)	↑ (Gödecke et al. 1999)			
Exercise capacity	↔ (Garry et al. 1998)	↓ (Merx et al. 2005)			
Shift from fatty acid to glucose		↑ (Flögel et al. 2005)			
Lactate utilization	↑ (Meeson et al. 2001)				
RER		↑ rest and exercise (Merx et al. 2005)<br>↔ rest (Christen et al. 2022)	↔ rest on HFD (Ono-Moore et al. 2021)		
Upregulated/downregulated genes in the heart	4/0 qPCR (Meeson et al. 2001)	6/9 qPCR (Schlieper et al. 2004)<br>0/3 qPCR (Flögel et al. 2005)<br>64/52 microarray (Molojavyi et al. 2010)			0/0 RNAseq

Supp. Table 2 Guide RNA (gRNA) sequences and the used oligonucleotides. The underlined nucleotides (TAGGG in the 5' end for sense or AAAC in the 5' and C in the 3' end for antisense) were added to facilitate cloning into pDR274. All sequences are shown in the 5' to 3' direction

**gRNA Name**	**gRNA (5'-3')**	**Oligonucleotides for cloning into pDR274**	
		**Sense (5'-3')**	**Antisense (5'-3')**
#1 gRNAmbEx1-096(-)	GTCGTTAAACAAGCGATGTA	TAGGGCGTTAAACAAGCGATGTA	AAACTACATCGCTTGTTTAACGC
#2 gRNAmbEx1-065(-)	GGGGCTTGTGTGAACACGTC	TAGGGGGCTTGTGTGAACACGTC	AAACGACGTGTTCACACAAGCCC
#3 gRNAmbEx1-046(-)	CACACCTGTCTTCAATATCG	TAGGGCACCTGTCTTCAATATCG	AAACCGATATTGAAGACAGGTGC
#4 gRNAmbEx1-008(-)	GATCAGCCATCGTCTGTCTG	TAGGGTCAGCCATCGTCTGTCTG	AAACCAGACAGACGATGGCTGAC
#5 gRNAmbEx1-003(+)	ATGTTTCCTCAGACAGACGA	TAGGGGTTTCCTCAGACAGACGA	AAACTCGTCTGTCTGAGGAAACC
#6 gRNAmbEx1+028(+)	TGATCTGGTTCTGAAGTGCT	TAGGGATCTGGTTCTGAAGTGCT	AAACAGCACTTCAGAACCAGATC
#7 gRNAmbEx1+040(+)	GAAGTGCTGGGGAGCCGTGG	TAGGGAGTGCTGGGGAGCCGTGG	AAACCCACGGCTCCCCAGCACTC
#8 gRNAmbEx1+043(-)	AGCCGCATAATCGGCCTCCA	TAGGGCCGCATAATCGGCCTCCA	AAACTGGAGGCCGATTATGCGGC
#9 gRNAmbEx1+059(+)	GAGGCCGATTATGCGGCTAA	TAGGGGGCCGATTATGCGGCTAA	AAACTTAGCCGCATAATCGGCCC
#10gRNAmbEx1+062(+)	GCCGATTATGCGGCTAACGG	TAGGGCGATTATGCGGCTAACGG	AAACCCGTTAGCCGCATAATCGC
#11 gRNAmbEx2+077(+)	GGGTGATCTGGCGGGAAGCC	TAGGGGTGATCTGGCGGGAAGCC	AAACGGCTTCCCGCCAGATCACC
#12 gRNAmbEx2+080(+)	TGATCTGGCGGGAAGCCCGG	TAGGGATCTGGCGGGAAGCCCGG	AAACCCGGGCTTCCCGCCAGATC
#13 gRNAmbEx2+083(+)	TCTGGCGGGAAGCCCGGCGG	TAGGGTGGCGGGAAGCCCGGCGG	AAACCCGCCGGGCTTCCCGCCAC
#14 gRNAmbEx2+086(+)	GGCGGGAAGCCCGGCGGTGG	TAGGGCGGGAAGCCCGGCGGTGG	AAACCCACCGCCGGGCTTCCCGC
#16 gRNAmbEx2+093(+)	AGCCCGGCGGTGGCGGCTCA	TAGGGCCCGGCGGTGGCGGCTCA	AAACTGAGCCGCCACCGCCGGGC
#17 gRNAmbEx2+111(-)	TTCGCCCAGCTTCTTGAGCA	TAGGGCGCCCAGCTTCTTGAGCA	AAACTGCTCAAGAAGCTGGGCGC
#18 gRNAmbEx2+116(+)	AGCGACCGTGCTCAAGAAGC	TAGGGCGACCGTGCTCAAGAAGC	AAACGCTTCTTGAGCACGGTCGC
#19 gRNAmbEx2+117(+)	GCGACCGTGCTCAAGAAGCT	TAGGGGACCGTGCTCAAGAAGCT	AAACAGCTTCTTGAGCACGGTCC
#21 gRNAmbEx2+138(+)	GGCGAACTGCTGAAGGCCAA	TAGGGCGAACTGCTGAAGGCCAA	AAACTTGGCCTTCAGCAGTTCGC

Supp. Table 3 Oligonucleotides used as primers in PCR and Sanger sequencing. All sequences are shown in the 5' to 3' direction

Oligo name	Sequence 5'-3'
mbEx1-1240_f	GAGGTCGTTTAAGGTCAGAAGTG
mbEx1+482_r	GAAATTAAACACTAGTGGGCGG
mbEx1+728_r	GGGGAAGAGCTTCAGAGTGTC
mbEx2-202_f	AATAGCTAAACAGCAACTGGGG
mbEx2+272_r	TCAATGTGGATGCTTAGTCGAG
mbEx1-282_f	CCAATAGATGCCAGCAACGA
mbEx2+353_r	ACATACTGTGAAAATTTGGGGG
smbEx1-205_f	GAGCAAGAGTAAACGTTGAGG
mbEx2+266_r	TGGATGCTTAGTCGAGCAGTG

Supp. Table 4 *In vivo* efficiency of the individual guide RNAs (gRNA) as determined by TIDE or ICE analyses

Name	*in vivo* efficiency (%)		Name	*in vivo* efficiency (%)		Name	*in vivo* efficiency (%)	
	TIDE	ICE		TIDE	ICE		TIDE	ICE
gRNA1	9.5	0	gRNA8	5.8	0	gRNA16	24.25	9
gRNA2	5.1	0	gRNA9	10.7	0	gRNA17	29.1	NA
gRNA3	3.8	0	gRNA10	15.4	0	gRNA18	22.2	9
gRNA4	9.1	0	gRNA11	12.8	NA	gRNA19	31.7	NA
gRNA5	7.6	0	gRNA12	80.9	85	gRNA21	75.4	NA
gRNA6	24.4	3	gRNA13	73.1	84			
gRNA7	66.4	73	gRNA14	13.8	4			

Supp. Table 5 Genomic mutations in *mb*$^{\text{Auzf13.2}}$, *mb*$^{\text{Auzf13.3}}$, and *mb*$^{\text{Auzf13.6}}$. Dashes indicates insertion if in wild-type sequence and deletions if in mutant sequence. Italic and lowercase lettering indicates inserted sequence in the *mb*$^{\text{Auzf13.2}}$ line

	Exon 1	Intron 1	Exon 2	Exon2	Exon 2
Chr1	55141060..55141069	55141386..55141395	55141826..55141831	55141810..55141819	55141916..55141925
wild-type	GAGCCGTGGA	CTAATTCAGA	CTCATG	AGCCC-----GGCGG	ACGCCAACAT
mb$^{\text{Auzf13.2}}$				AGCC*ggtggt*GGCGG	
mb$^{\text{Auzf13.3}}$		CTAAT-----	---ATG		
mb$^{\text{Auzf13.6}}$	GAGCC-----	----------	------		-----AACAT

Supp. Table 6 mRNA sequence expected to be transcribed in wild-type, $mb^{\mathrm{Auzf13.2}}$, $mb^{\mathrm{Auzf13.3}}$, and $mb^{\mathrm{Auzf13.6}}$. Italic and lowercase lettering indicates inserted sequence in the $mb^{\mathrm{Auzf13.2}}$. Bold lettering indicates termination codons

	Exon1	Exon2	Exon3
wild-type	ATGGCTGATCATGA<br>TCTGGTTCTGAAGT<br>GCTGGGGAGCCGTG<br>GAGGCCGATTATGC<br>GGCTAACGGAGGAG<br>AAGTTCTCAACCG	TCTGTTCAAGGAGTATCCAGACACTCTGAAGCTCTTC<br>CCCAAGTTTTCTGGGATTTCTCAGGGTGATCTGGCGG<br>GAAGCCCGGCGGTGGCGGCTCATGGAGCGACCGTGCT<br>CAAGAAGCTGGGCGAACTGCTGAAGGCCAAAGGAGAC<br>CACGCTGCCCTGCTCAAACCACTGGCCAATACACACG<br>CCAACATTCACAAAGTGGCCCTCAACAACTTCAGG	CTGATCACCGAGGTGCTGGTGAAA<br>GTGATGGCCGAAAAGGCGGGTCTG<br>GACGCGGCCGGTCAAGGCGCCCTG<br>AGGCGGGTCATGGACGCGGTCATC<br>GGGGACATCGACGGATACTACAAG<br>GAGATCGGATTTGCCGGT**TAA**
$mb^{\mathrm{Auzf13.2}}$	ATGGCTGATCATGA<br>TCTGGTTCTGAAGT<br>GCTGGGGAGCCGTG<br>GAGGCCGATTATGC<br>GGCTAACGGAGGAG<br>AAGTTCTCAACCG	TCTGTTCAAGGAGTATCCAGACACTCTGAAGCTCTTC<br>CCCAAGTTTTCTGGGATTTCTCAGGGTGATCTGGCGG<br>GAAGCC*ggtggt*GGCGGTGGCGGCTCATGGAGCGACC<br>GTGCTCAAGAAGCTGGGCGAACTGC**TGA**AGGCCAAAG<br>GAGACCACGCTGCCCTGCTCAAACCACTGGCCAATAC<br>ACACGCCAACATTCACAAAGTGGCCCTCAACAACTTC<br>AGG	CTGATCACCGAGGTGCTGGTGAAA<br>GTGATGGCCGAAAAGGCGGGTCTG<br>GACGCGGCCGGTCAAGGCGCCCTG<br>AGGCGGGTCATGGACGCGGTCATC<br>GGGGACATCGACGGATACTACAAG<br>GAGATCGGATTTGCCGGT**TAA**
$mb^{\mathrm{Auzf13.3}}$	ATGGCTGATCATGA<br>TCTGGTTCTGAAGT<br>GCTGGGGAGCCGTG<br>GAGGCCGATTATGC<br>GGCTAACGGAGGAG<br>AAGTTCTCAACCG	skipped	C**TGA**TCACCGAGGTGCTGGTGAAA<br>GTGATGGCCGAAAAGGCGGGTCTG<br>GACGCGGCCGGTCAAGGCGCCCTG<br>AGGCGGGTCATGGACGCGGTCATC<br>GGGGACATCGACGGATACTACAAG<br>GAGATCGGATTTGCCGGT**TAA**
$mb^{\mathrm{Auzf13.6}}$	ATGGCTGATCATGA<br>TCTGGTTCTGAAGT<br>GCTGGGGAGCCAAC<br>ATTCACAAAGTGGC<br>CCTCAACAACTTCA<br>GG		CTGATCACCGAGGTGCTGGTGAAA<br>GTGATGGCCGAAAAGGCGGGTCTG<br>GACGCGGCCGGTCAAGGCGCCCTG<br>AGGCGGGTCATGGACGCGGTCATC<br>GGGGACATCGACGGATACTACAAG<br>GAGATCGGATTTGCCGGT**TAA**

Supp. Table 7 Amino acid sequences resulting from translation of mRNA from wild-type, *mb*$^{Auzf13.2}$, *mb*$^{Auzf13.3}$, and *mb*$^{Auzf13.6}$. Changes in primary structure are indicated by italic, and stop codons are indicated by an asterisk. His-60 (E7), involved in heme binding and only present in protein translated from wild-type mRNA, is shown in bold

	Mb primary structure
wild-type	MADHDLVLKCWGAVEADYAANGGEVLNRLFKEYPDTLKLFPKFSGISQGDLAGSPAVAA**H**GATVLKKLGELLKAKGDHAAL<br>LKPLANTHANIHKVALNNFRLITEVLVKVMAEKAGLDAAGQGALRRVMDAVIGDIDGYYKEIGFAG*
mb$^{Auzf13.2}$	MADHDLVLKCWGAVEADYAANGGEVLNRLFKEYPDTLKLFPKFSGISQGDLAGS_RWWRWRLMERPCSRSWANC*_
mb$^{Auzf13.3}$	MADHDLVLKCWGAVEADYAANGGEVLNR*
mb$^{Auzf13.6}$	MADHDLVLKCWGA--<br>---------NIHKVALNNFRLITEVLVKVMAEKAGLDAAGQGALRRVMDAVIGDIDGYYKEIGFAG*

Supp. Table 8 Differentially expressed genes as determined by DESeq2

GeneID	BaseMean	log2FC	P_{adj}	Chromosome	GeneName
ENSDARG00000031952	210408.28	-5.20	0	1	mb
ENSDARG00000037403	168.16	-1.48	0.00	15	hspa8b
ENSDARG00000074052	32.31	-1.23	0.00	1	si:ch211-281g13.5
ENSDARG00000074136	32.22	-1.02	0.00	1	slc47a2.2
ENSDARG00000103044	30.44	-0.97	0.00	NA	ASS1
ENSDARG00000043431	29.98	-0.92	0.00	1	b3gntl1
ENSDARG00000078389	241.97	-0.91	0.00	13	ifi46
ENSDARG00000099447	19.29	-0.86	0.02	10	pcdh1g18
ENSDARG00000090557	6.92	-0.84	0.00	1	MFAP4
ENSDARG00000102178	42.64	-0.83	0.03	7	emilin2b
ENSDARG00000013087	595.06	-0.83	0.02	11	ndrg3a
ENSDARG00000095697	191.11	-0.80	0.01	1	CR774194.1
ENSDARG00000069472	332.02	-0.77	0.04	10	chsy3
ENSDARG00000099842	108.60	-0.76	0.04	3	CU138533.1
ENSDARG00000088251	214.89	-0.71	0.03	15	lgals17
ENSDARG00000043663	253.35	-0.70	0.00	10	faub
ENSDARG00000055064	2243.11	-0.69	0.01	21	prdx5
ENSDARG00000028000	1092.87	-0.61	0.02	24	pfkpa
ENSDARG00000090980	535.38	-0.58	0.02	6	apof
ENSDARG00000029764	1857.48	-0.57	0.03	10	mef2ca
ENSDARG00000086471	2879.42	-0.49	0.01	23	pkig
ENSDARG00000056915	670.47	-0.42	0.04	10	si:ch211-237l4.6
ENSDARG00000041589	11524.19	-0.40	0.00	1	adprhl1
ENSDARG00000053136	8332.68	-0.40	0.04	4	b2m
ENSDARG00000109848	899.31	-0.40	0.01	1	FO681286.1
ENSDARG00000036764	829.95	-0.38	0.04	19	hax1
ENSDARG00000013475	7689.39	-0.37	0.01	1	cct4
ENSDARG00000076939	258.10	0.53	0.04	13	znf451
ENSDARG00000078430	634.67	0.57	0.00	10	tiam1a
ENSDARG00000030844	110.66	0.68	0.02	20	klf11a
ENSDARG00000054058	259.50	0.70	0.04	6	h1-10
ENSDARG00000009136	955.67	0.70	0.01	13	tp53bp2a
ENSDARG00000015156	209.31	0.75	0.03	11	fbln2
ENSDARG00000002656	81.60	0.79	0.02	20	stxbp5a
ENSDARG00000097897	421.68	0.79	0.00	6	sgip1a
ENSDARG00000092813	79.04	0.80	0.04	2	fndc7rs4
ENSDARG00000104458	140.30	0.80	0.02	10	rnasekb
ENSDARG00000070951	150.45	0.82	0.01	6	hmga1b
ENSDARG00000029457	60.46	0.82	0.01	22	cacna1sa
ENSDARG00000094695	56.64	0.82	0.01	3	si:dkey-204f11.64
ENSDARG00000098834	124.83	0.83	0.01	16	sox4b
ENSDARG00000079564	5753.64	0.86	0.01	2	myh7
ENSDARG00000099500	36.12	0.88	0.01	19	clec3bb
ENSDARG00000075727	241.23	0.92	0.00	14	map1lc3cl
ENSDARG00000101818	64.37	0.93	0.01	7	BX294178.1
ENSDARG00000098880	236.45	0.95	0.00	18	pclob
ENSDARG00000017780	103.68	1.00	0.00	2	rorcb
ENSDARG00000068934	442.53	1.02	0.00	13	cyp1b1
ENSDARG00000034420	104.65	1.15	0.00	7	irx6a

References

Christen, L., H. Broghammer, I. Rapöhn, K. Möhlis, C. Strehlau, A. Ribas-Latre, C. Gebhardt, L. Roth, K. Krause, K. Landgraf, A. Körner, K. Rohde-Zimmermann, A. Hoffmann, N. Klöting, A. Ghosh, W. Sun, H. Dong, C. Wolfrum, T. Rassaf, U. B. Hendgen-Cotta, M. Stumvoll, M. Blüher, J. T. Heiker, and J. Weiner. 2022. 'Myoglobin-mediated lipid shuttling increases adrenergic activation of brown and white adipocyte metabolism and is as a marker of thermogenic adipocytes in humans', *Clin Transl Med*, 12: e1108. https://doi.org/10.1002/ctm2.1108.

Flögel, U., T. Laussmann, A. Gödecke, N. Abanador, M. Schäfers, C. D. Fingas, S. Metzger, B. Levkau, C. Jacoby, and J. Schrader. 2005. 'Lack of myoglobin causes a switch in cardiac substrate selection', *Circ Res*, 96: e68-75. https://doi.org/10.1161/01.RES.0000165481.36288.d2.

Garry, D. J., G. A. Ordway, J. N. Lorenz, N. B. Radford, E. R. Chin, R. W. Grange, R. Bassel-Duby, and R. S. Williams. 1998. 'Mice without myoglobin', *Nature*, 395: 905-8. https://doi.org/10.1038/27681.

Grange, R. W., A. Meeson, E. Chin, K. S. Lau, J. T. Stull, J. M. Shelton, R. S. Williams, and D. J. Garry. 2001. 'Functional and molecular adaptations in skeletal muscle of myoglobin-mutant mice', *Am J Physiol Cell Physiol*, 281: C1487-94. https://doi.org/10.1152/ajpcell.2001.281.5.C1487.

Gödecke, A., U. Flögel, K. Zanger, Z. Ding, J. Hirchenhain, U. K. Decking, and J. Schrader. 1999. 'Disruption of myoglobin in mice induces multiple compensatory mechanisms', *Proc Natl Acad Sci U S A*, 96: 10495-500. https://doi.org/10.1073/pnas.96.18.10495.

Gödecke, A., A. Molojavyi, J. Heger, U. Flögel, Z. Ding, C. Jacoby, and J. Schrader. 2003. 'Myoglobin protects the heart from inducible nitric-oxide synthase (iNOS)-mediated nitrosative stress', *J Biol Chem*, 278: 21761-6. https://doi.org/10.1074/jbc.M302573200.

Hendgen-Cotta, U. B., S. Esfeld, C. Coman, R. Ahrends, L. Klein-Hitpass, U. Flögel, T. Rassaf, and M. Totzeck. 2017. 'A novel physiological role for cardiac myoglobin in lipid metabolism', *Sci Rep*, 7: 43219. https://doi.org/10.1038/srep43219.

Hendgen-Cotta, U. B., M. W. Merx, S. Shiva, J. Schmitz, S. Becher, J. P. Klare, H. J. Steinhoff, A. Goedecke, J. Schrader, M. T. Gladwin, M. Kelm, and T. Rassaf. 2008. 'Nitrite reductase activity of myoglobin regulates respiration and cellular viability in myocardial ischemia-reperfusion injury', *Proc Natl Acad Sci U S A*, 105: 10256-61. https://doi.org/10.1073/pnas.0801336105.

Mammen, P. P., S. B. Kanatous, I. S. Yuhanna, P. W. Shaul, M. G. Garry, R. S. Balaban, and D. J. Garry. 2003. 'Hypoxia-induced left ventricular dysfunction in myoglobin-deficient mice', *Am J Physiol Heart Circ Physiol*, 285: H2132-41. https://doi.org/10.1152/ajpheart.00147.2003.

Meeson, A. P., N. Radford, J. M. Shelton, P. P. Mammen, J. M. DiMaio, K. Hutcheson, Y. Kong, J. Elterman, R. S. Williams, and D. J. Garry. 2001. 'Adaptive mechanisms that preserve cardiac function in mice without myoglobin', *Circ Res*, 88: 713-20. https://doi.org/10.1161/hh0701.089753.

Merx, M. W., A. Gödecke, U. Flögel, and J. Schrader. 2005. 'Oxygen supply and nitric oxide scavenging by myoglobin contribute to exercise endurance and cardiac function', *Faseb j*, 19: 1015-7. https://doi.org/10.1096/fj.04-2886fje.

Molojavyi, Andrei, Antje Lindecke, Annika Raupach, Sarah Moellendorf, Karl Köhrer, and Axel Gödecke. 2010. 'Myoglobin-deficient mice activate a distinct cardiac gene expression program in response to isoproterenol-induced hypertrophy', *Physiological Genomics*, 41: 137-45. https://doi.org/10.1152/physiolgenomics.90297.2008.

Ono-Moore, K. D., I. M. Olfert, J. M. Rutkowsky, S. V. Chintapalli, B. J. Willis, M. L. Blackburn, D. K. Williams, J. O'Reilly, T. Tolentino, K. C. K. Lloyd, and S. H. Adams. 2021. 'Metabolic physiology and skeletal muscle phenotypes in male and female myoglobin knockout mice', *Am J Physiol Endocrinol Metab*, 321: E63-e79. https://doi.org/10.1152/ajpendo.00624.2020.

Schlieper, Georg, Jie-Hoon Kim, Andrei Molojavyi, Christoph Jacoby, Tim Laussmann, Ulrich Flögel, Axel Gödecke, and Jurgen Schrader. 2004. 'Adaptation of the myoglobin knockout mouse to hypoxic stress', *American journal of physiology. Regulatory, integrative and comparative physiology*, 286: R786-92. https://doi.org/10.1152/ajpregu.00043.2003.

Vlecken, D. H., J. Testerink, E. B. Ott, P. A. Sakalis, R. T. Jaspers, and C. P. Bagowski. 2009. 'A critical role for myoglobin in zebrafish development', *Int J Dev Biol*, 53: 517-24. https://doi.org/10.1387/ijdb.082651dv.
